# Subjective Verticality Is Disrupted by Astigmatic Visual Distortion in Older People

**DOI:** 10.1167/iovs.61.4.12

**Published:** 2020-04-15

**Authors:** David B. Elliott, Alexander A. Black, Joanne M. Wood

**Affiliations:** 1 School of Optometry & Vision Science, University of Bradford, Bradford, United Kingdom; 2 School of Optometry & Vision Science, Queensland University of Technology, Brisbane, Australia

**Keywords:** visual vertical, magnification, postural stability, dizziness, astigmatism

## Abstract

**Purpose:**

There is little research evidence to explain why older adults have more problems adapting to new spectacles incorporating astigmatic changes than younger adults. We tested the hypothesis that astigmatic lenses oriented obliquely would lead to errors in verticality perception that are greater for older than younger adults.

**Methods:**

Participants included 12 young (mean ± SD age 25.1 ± 5.0 years) and 12 older (70.2 ± 6.3 years) adults with normal vision. Verticality perception was assessed using a computer-based subjective visual vertical (SVV) task, under static and dynamic (in the presence of a moving peripheral distractor) conditions and when viewing targets through the near refractive correction (control condition), and two forms of astigmatic lenses oriented in the vertical, horizontal, and oblique meridians.

**Results:**

The older group demonstrated much greater dynamic SVV errors (e.g., 3.4° for the control condition) than the younger group (1.2°, *P* = 0.002), larger errors with vertical and horizontal astigmatic lenses (older group 4.1°and 5.2° for toric and magnifier lenses vs. younger group 1.2° and 1.4°, respectively, *P* < 0.001), and a larger influence of the oblique astigmatic lenses (older group 5.6° vs. younger group 2.1°, *P*<0.001).

**Conclusions:**

Astigmatic lenses produced little or no errors in SVV in young adults, but large static and dynamic SVV errors in older adults. This indicates a greater reliance on visual input with increased age for SVV, and helps explain why oblique astigmatic refractive corrections can cause dizziness in older patients and why they report greater difficulties adapting to new spectacles with astigmatic changes.

There is substantial experience-based clinical evidence that older adults adapt less well to new spectacles than younger adults,^1^^–^^3^ and this is confirmed in studies of patients who return to complain about difficulties with new spectacles.^4^ However, there is little research evidence to explain why this occurs. Indeed, the main research finding indicating that refractive changes can be a problem for older patients was a serendipitous finding from a randomized controlled trial of eye examinations and their effects on fall rate.^5^ The hypothesis of this randomized controlled trial was that updated refractive corrections and cataract surgery would reduce fall rate, but the opposite was found and large changes in refractive correction (≥ ±0.75 Diopters Sphere (DS) or Diopters Cylinder (DC), axis changes of ≥10° up to 0.75 DC and ≥5° for 0.75 DC+, any prism change or an introduced anisometropia of ≥0.75 DS) led to an *increased* fall rate (74% vs. 52% with smaller correction changes).^5^ Similar findings were found for the effects of changes in refractive correction on fall rates following cataract surgery.^6^

Oblique astigmatic changes are particularly known to cause problems for older adults.^1^^–^^3^^,^^7^ They can produce symptoms of tilted walls, sloping floors, and elongated shapes of objects,^1^^,^^2^ as well as dizziness in older patients postcataract surgery.^7^ These perceptual changes are due to the distortive effects of astigmatic lenses, particularly when orientated at an oblique axis.^8^^,^^9^ We hypothesized that the effects of oblique astigmatic lens changes are likely to be due to disruption in verticality perception, which is important for the maintenance of upright stance, postural stability and gait, and avoidance of dizziness.^10^^–^^15^

Perception of verticality is typically assessed using subjective visual vertical (SVV), where the observer is asked to align a rod vertically with all other visual cues removed (e.g., the task is performed in the dark and viewed through a circular tube).^11^ SVV is widely used as a clinical test for patients with vestibular lesions,^12^^,^^13^ and abnormal SVV has been linked with poor postural stability and falls and symptoms of dizziness.^10^^–^^15^ Dynamic SVV, where the vertical alignment task is performed in the presence of a moving peripheral distractor, is used to determine the influence of visual input on verticality perception.^16^ For example, dynamic SVV has been used to demonstrate the greater visual dependence of patients with visual vertigo for verticality perception.^10^^,^^15^

We hypothesized that astigmatic lenses in the oblique meridian could disrupt verticality perception and static SVV and be particularly disruptive to dynamic SVV, given that it is more strongly influenced by visual input. We further hypothesized that the effect of astigmatic lenses on SVV would be greater in older than young adults, given that they adapt less well to astigmatic refractive changes,^1^^–^^3^ and may be more reliant on visual input for verticality perception than younger adults.^17^ We used two types of astigmatic lenses in our study: toric lenses (i.e., astigmatism-correcting spectacle lenses), which cause both meridional magnification and can induce blur, and meridional magnifier lenses, which do not induce any blur. This enabled us to differentiate between the effects of meridional magnification and its direction on SVV with those of astigmatic blur.^18^

## Methods

### Participants

Twenty-four participants were recruited and included 12 young (18–30 years; mean ± SD age, 25.1 ± 5.0 years) and 12 older (60+ years; mean ± SD age, 70.2 ± 6.3 years) adults who were free of ocular disease and had normal visual acuity (better than 0.20 logMAR or 20/30 for the older group, and better than 0.00 logMAR or 20/20 for the younger group).

Exclusion criteria included self-reported vestibular or ocular disease, visual vertigo, dizziness in the last 3 months and/or poor balance control, and/or taking medications known to affect dizziness and balance control, such as sedatives and antidepressants. The study gained ethical approval from Queensland University of Technology Human Research Ethics Committee and the tenets of the Declaration of Helsinki were followed. All participants gave written informed consent.

### Procedure

All measurements were made monocularly using the participants’ dominant eye as determined using a simple pointing test, or the participants' preferred eye (if there was no obvious ocular dominance). Measurements of static and dynamic SVV and visual acuity were made using the participants’ habitual refractive correction (as measured using a lens meter), plus a near working distance lens placed in a trial frame using trial lenses. We measured SVV in the presence and absence of astigmatic lenses in the vertical, horizontal, and oblique meridians in young and older adults. Two types of astigmatic lenses were used: (1) toric lenses of +1.00 DS/–2.00 DC, which produced both astigmatic blur and altered magnification of approximately –2% along one meridian. (2) Custom-made magnifying cylindrical lenses with zero magnification along one meridian and –3.2% along the perpendicular meridian. These lenses do not blur vision, so that any effects on SVV would be due to meridional magnification.^9^ These astigmatic lenses create an offset to verticality perception (or declination error) when astigmatic lenses are placed obliquely in front of the eye.^19^ The vertical declination error δv can be calculated from the equation δv = –0.29f sin 2θ, where f is the difference in magnification between the two astigmatic meridians, and θ indicates the orientation of the lens axis, which is the meridian of the lens with zero power.^18^ When oriented horizontally (θ = 0° or 180°) or vertically (θ = 90°), δv = 0°. When oriented obliquely at 45°, δv = +0.93° (meridional magnifier lenses) and δv = +0.58° (toric lenses), and the lenses make a vertical straight line appear to have rotated by approximately 0.6° to 1.0° in a clockwise direction.

The following vision conditions were tested in a random order (with x indicating the axis in degrees, and with the axis meridian of the cylinder and meridional magnifier having zero power):

Toric lenses of +1.00/–2.00 × 90
Toric lenses of +1.00/–2.00 × 180
Toric of +1.00/–2.00 × 45
Meridional magnifier lens of –3.2% x 90.
Meridional magnifier lens of –3.2% x 180
Meridional magnifier lens of –3.2% x 45
Control (no additional lenses).

Visual acuity was measured using the computer-based Freiburg Vision Test (FrACT; www.michaelbach.de/fract/index.html),^20^ as the effects of astigmatic lenses on visual acuity have been shown to have poor repeatability with standard letter charts, due to variable influences of diplopia and aberrations.^21^ Single high-contrast Landolt Cs were presented on a computer screen at 6 m, and participants had to indicate the location of the gap in the letter C using an eight-alternative forced-choice procedure. Letter size changes were based on participant responses using the Best PEST (Parameter Estimation by Sequential Testing) psychophysical procedure, and the visual acuity threshold was determined as the steepest point of the psychometric function gained from all the data.^20^ A constant slope on a logarithmic acuity scale was assumed. As most letters presented were near threshold and difficult to see, a letter approximately four times larger than the current threshold estimate was presented every sixth trial to maintain participants’ attention. Participants were also instructed that many of the letters would be difficult to see, and to guess if they were unsure.

Static and dynamic SVV were measured on a computer screen with a system developed and reported previously.^15^ Our pilot studies with older participants indicated that one instructional trial of eight repetitions (for both static and dynamic SVV) was sufficient training, and that seven vision conditions, each with eight repetitions, provided data with minimal fatigue effects. Participants were seated in front of the screen in a darkened room, with their head upright and positioned in a chin and head rest so that the vertical axis of the head was aligned with earth vertical defined by gravity. A tubular viewing cone between the head rest and screen blocked extraneous visual orientation cues. The diameter and length of the cone at the participants’ eyes were 25 and 50 cm, respectively.

Static SVV was measured using a visual stimulus comprising a luminous white 9.5-cm rod on a black background. Participants were instructed to align the rod to their perceived vertical using a roller mouse, which had an accuracy of 0.1°. This was repeated eight times, with a random starting orientation of ±20° from vertical. The rod tilt for each trial was recorded as the difference in degrees between true vertical (as defined by a vertically aligned rod on the screen with 0° error) and the subjects’ final placement of the rod, and a mean value was taken from the eight measurements, with positive values indicating a clockwise tilt. SVV data as a function of testing time suggested no obvious adaptation to the lenses over the period of testing.

**Figure 1.**
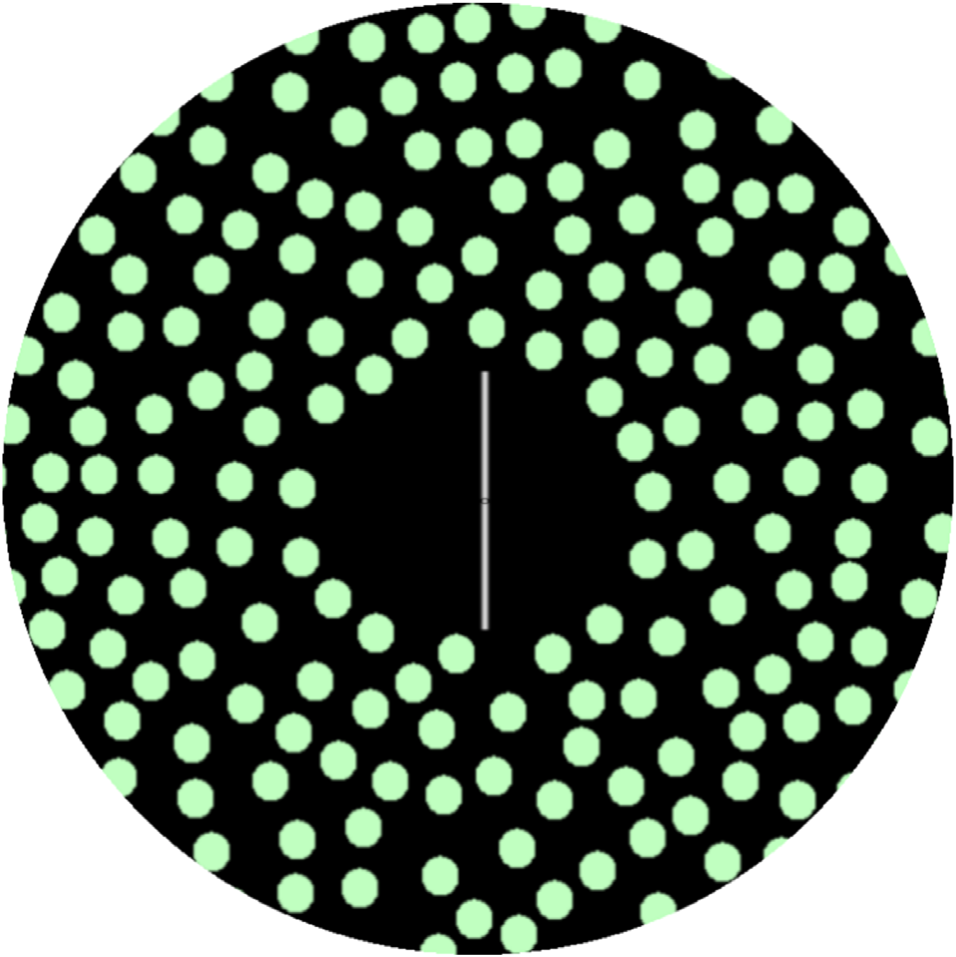
The target for the dynamic SVV. The circular discs moved clockwise at 30°/s. The task was to align the rod vertically from a starting orientation of random tilt.

Dynamic SVV measurements were taken using the same procedure, but the visual stimulus consisted of a luminous white 6-cm rod on a black background, with the peripheral ring comprising 220 off-white dots, each 8 mm (1.5° of visual field) in diameter, randomly distributed on the black background (Fig. 1). The dots rotated clockwise at 30°/s.^10^^,^^15^ In dynamic SVV, the vertical line appears to tilt in the opposite direction to the movement of the peripheral stimulus,^16^ so that when participants are asked to orientate the line vertically there is an error in the direction of the movement of the peripheral stimulus. The rotation direction and oblique magnifier/toric lens orientation were selected so that their potential effects would be additive, that is, a clockwise rotation provides a clockwise verticality error and would add to a clockwise verticality error potentially provided by negative magnification with the axis at an oblique 45° orientation.

### Data Analyses

Statistical analyses were performed using SPSS version 23.0 (IBM Corporation, Armonk, NY, USA), and the level of significance was set at *P* < 0.05. Group differences (younger vs. older) in visual acuity in the control condition (i.e., no additional lenses) were analyzed with an independent samples *t*-test. Differences in visual acuity between the control, toric, and meridional magnifier lenses were assessed using a mixed-design ANOVA, which included between-group effects and lens condition within-effects. The Greenhouse-Geisser correction was applied to the degrees of freedom if sphericity was violated.

Separate linear regression models with generalized estimating equations (GEE) for repeated measures were used to ascertain changes in the static and dynamic SVV responses using an exchangeable correlation structure. Each model included the following factors: (1) age group (younger vs. older), (2) lens type (control, toric, or meridional magnifier), and (3) astigmatic lens orientation (none, vertical, horizontal, or oblique), and all two- and three-way interactions. Any significant interactions were further tested to understand the nature and direction of these relationships.

## Results

Visual acuity for the two age groups with the toric and meridional magnifier lenses are shown in Table 1. The younger group had significantly better visual acuity than the older group (–0.14 ± 0.07 vs. –0.07 ± 0.08 logMAR, Snellen equivalents ∼20/15 vs. 20/17; t(22) = –2.41, *P* = 0.025). These visual acuities are typical for healthy eyes in young and older age groups,^22^ with both means being better than 20/20.

**Table 1.** Mean (with SD in brackets) of Visual Acuity (logMAR) for the Young and Older Groups, with Control (no additional lenses) and Additional Toric and Meridional Magnifier Lenses at Vertical, Horizontal, and Oblique Orientations

	Group	
	Young (*n* = 12)	Older (*n* = 12)
Control	–0.14 (0.07)	–0.07 (0.08)
Vertical toric	0.31 (0.16)	0.28 (0.12)
Horizontal toric	0.27 (0.12)	0.33 (0.16)
Oblique toric	0.33 (0.11)	0.35 (0.14)
Vertical meridional magnifier	–0.09 (0.10)	–0.01 (0.16)
Horizontal meridional magnifier	–0.08 (0.10)	0.05 (0.17)
Oblique meridional magnifier	–0.07 (0.09)	0.02 (0.19)

The 2-way ANOVA for visual acuity for all vision conditions showed a significant main effect of lens type (F_3.5,76.1_ = 74.9, *P* < 0.001). Visual acuity was reduced (*P* < 0.001) by the meridional magnifier lenses compared with the control lens but the effect was clinically small at approximately 0.07 logMAR (less than one line of letters) and did not differ between orientations (*P* = 0.99). Visual acuity through the toric lenses was reduced by approximately 0.40 logMAR (four lines), which represents a clinically significant effect compared with the control (*P* < 0.001) and did not differ between orientations (*P* = 0.99). Thus visual acuity loss was over four times greater with the toric compared with the meridional magnifier lenses (Table 1). There was no significant main effect of age group (F_1,22_ = 3.15, *P* = 0.09) and no significant interaction (F_3.5,76.1_ = 1.3, *P* = 0.29), indicating that the effects of the toric and meridional magnifier lenses on visual acuity were similar for both age groups.

**Figure 2.**
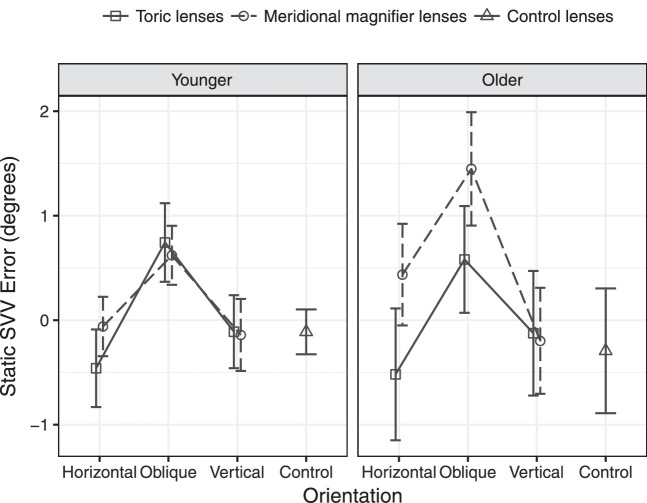
Static SVV errors as a function of age group, lens type, and orientation. *Error bars* represent SEM.

**Figure 3.**
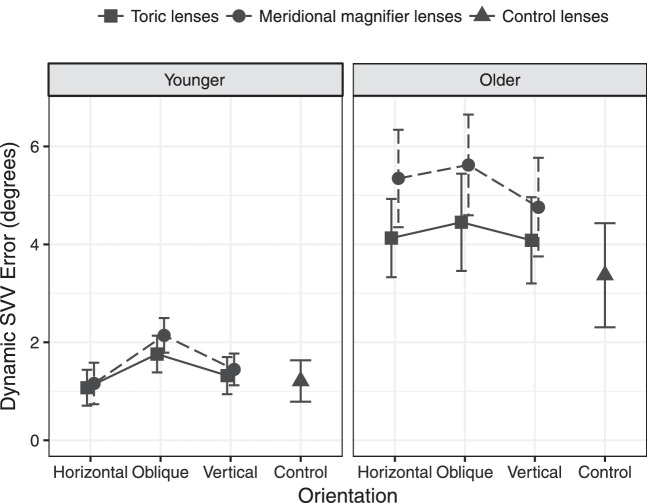
Dynamic SVV errors as a function of age group, lens type, and orientation. *Error bars* represent SEM.

The static and dynamic SVV results for the two age groups with the addition of the toric and meridional magnifier lenses are shown in Table 2 and Figures 2 and 3. Static SVV was typically between 0° to ±1° for all visual conditions for most of the young and older participants. This indicates that head alignment was accurate, and the vertical axis of the head was aligned with earth vertical defined by gravity. Dynamic SVV with the toric and meridional magnifier lenses was between +1° and +2° for young observers, but for older observers it was more variable and of larger magnitude, between approximately +3° and +6°.

**Table 2.** Mean and SD of Static and Dynamic SVV Errors in Degrees for the Young and Older Groups, with Control (no additional lenses) and Additional Toric or Meridional Magnifier Lenses at Horizontal, Vertical, and Oblique Meridians

	Static (˚)		Dynamic (˚)	
	Young	Older	Young	Older
Control	–0.1 (0.7)	–0.3 (2.1)	1.2 (1.5)	3.4 (3.7)
Horizontal toric	–0.5 (1.3)	–0.5 (2.2)	1.1 (1.3)	4.1 (2.8)
Horizontal meridional magnifier	–0.1 (1.0)	0.4 (1.7)	1.2 (1.5)	5.6 (3.5)
Vertical toric	–0.1 (1.2)	–0.1 (2.1)	1.3 (1.3)	4.1 (3.1)
Vertical meridional magnifier	–0.1 (1.2)	–0.2 (1.8)	1.5 (1.1)	4.8 (3.5)
Oblique toric	0.7 (1.3)	0.6 (1.8)	1.8 (1.3)	4.5 (3.4)
Oblique meridional magnifier	0.6 (1.0)	1.5 (1.9)	2.1 (1.2)	5.6 (3.6)

In the GEE models for the static SVV responses, there was a significant main effect of magnification orientation (Wald χ^2^ = 65.2, *P* < 0.001) and lens type (Wald χ^2^ = 4.1, *P* = 0.044), and a significant orientation x lens type interaction (Wald χ^2^ = 7.3, *P* = 0.026). No other main effects or interactions were observed (*P* > 0.05). To explore the significant interaction, pairwise comparisons were conducted separately by lens type, and showed that static SVV errors were significantly higher with all lenses (toric and meridional magnifier) when orientated at an oblique axis compared with the horizontal and vertical orientations (*P* < 0.001), and this effect was stronger for the meridional magnifier lenses compared with the toric lenses.

In the GEE models for the dynamic SVV, age group (Wald χ^2^ = 9.6, *P* = 0.002), orientation (Wald χ^2^ = 18.8, *P* < 0.001), and lens type (Wald χ^2^ = 11.0, *P* < 0.001) were significant factors in the model, with a significant interaction term of age x lens type (Wald χ^2^ = 5.0, *P* = 0.025). There were no other significant interactions (*P* > 0.05). Pairwise comparison in the full model showed that SVV errors were significantly higher for lenses at an oblique orientation compared with the horizontal and vertical orientations (*P* < 0.001), and this did not vary significantly with lens type (toric or meridional magnifier). To explore the significant interaction, the pairwise comparisons were examined separately by age group, and demonstrated that the meridional magnifier lenses increased SVV errors significantly for the older (*P* < 0.001), but not for the younger group (*P* = 0.23).

## Discussion

In this study we explored the effects of age, lens type (toric and meridional magnifier), and lens orientation (horizontal, vertical, and oblique) on perception of verticality under static and dynamic visual conditions. Our findings demonstrated that although static SVV did not vary as a function of age, dynamic SVV errors increased significantly, confirming a greater reliance on visual input for SVV in older people.^17^ We also showed that SVV in older adults was much more influenced by oastigmatic errors than in younger adults. Although for young adults, horizontal and vertical astigmatic lenses (of either type) had no effect on static and dynamic SVV, horizontal and vertical astigmatic lenses increased dynamic SVV errors in older adults, particularly for meridional magnifier lenses, which was further exacerbated with oblique astigmatic lenses. Toric lenses reduced visual acuity by more than four times that of the meridional magnifier lenses, but the latter had a greater effect on SVV in older adults, suggesting that SVV changes were caused by meridional magnification rather than blur.

The effects of the lenses on static SVV were similar for both the young and older age group and for all the control lenses, horizontal and vertical cylinders and astigmatic magnifiers. The magnitude of differences is typical of previously reported static SVV error values of ±1°.^15^ The increase in static SVV errors with the oblique cylinder and magnifier was similar for the younger and older groups, with mean increases of 0.8° and 1.1°, respectively. This is similar to their predicted declination errors linked to the degree of meridional magnification of approximately 0.58° and 0.93°.^1^^,^^18^

The dynamic SVV errors were much larger for the older age group and confirm the findings of Kobayashi et al.,^17^ although the age-related increases in errors found in the current study of approximately 0.05° per year are smaller than those reported by Kobayashi et al. (0.30° per year). This disparity may be due to stricter inclusion criteria for the level of vision for the older participants in the current study and differences in the SVV experimental setup, where Kobayashi et al. used a much larger peripheral region in a bowl apparatus.^17^ It has been suggested that the decrease in vestibular system function with age^23^ is associated with an increased contribution of visual input to SVV in older people.^17^

The young group showed no change in dynamic SVV errors with the horizontal and vertical astigmatic lenses and only small changes with the oblique astigmatic lenses of 0.6° and 0.9°, which is similar to optical theory predictions of declination error of 0.58° and 0.93°.^18^ However, the older age group showed significant increases in dynamic SVV errors with the horizontal and vertical astigmatic lenses (toric lenses, 0.7°; meridional magnifiers, 1.8°) relative to the control condition, with increases larger than the optical theory predictions of declination error of 0.58° and 0.93° for the oblique astigmatic lenses (toric, 1.1°; magnifier, 2.3° increase). The dynamic SVV errors caused by horizontal and vertical magnifier lenses in older people are likely to be due to interactions between the aberrations in the magnifier lenses and existing aberrations in the eye,^24^ and highlight the sensitivity of older adults to visual disruption of SVV.

The large increases in dynamic SVV error with the astigmatic lenses in the older group suggest that not only are older people more reliant on visual input for accurate SVV, but that their SVV can be disrupted to a greater extent than younger adults by changes to visual input. The increases in SVV error are likely to be clinically significant, given that the 2.2° increase with the oblique magnifier is approaching the magnitude of dynamic SVV difference of 3.4° between normal, asymptomatic patients of 2.96° relative to 6.35° for a group of patients with significant dizziness symptoms (Dizziness Handicap Inventory scores of 36–80) following recovery from acute vestibular neuritis.^15^ Young adults can adapt to oblique meridional magnification^9^ and relatively large cylindrical corrections (at all axes) in new spectacles^1^^–^^3^ in a few days. However, older patients are known to have much greater difficulty in adapting to cylindrical changes in new spectacles, particularly at oblique axes^1^^–^^3^ and in some cases are unable to adapt.^1^^–^^4^

## Conclusions

The fact that a large falls intervention study^5^ did not partially prescribe for large changes in refractive correction to their older, frail patients is indicative that many clinicians are unaware of the problems that can be caused by large refractive changes in older people. This is likely to be due to the fact that there is little research to explain why this difficulty in adapting occurs. The implications of the current study are that not only do older adults rely more on visual input for the perception of verticality,^17^ but that their perception is much more adversely affected by optical distortions, such as those provided by astigmatic changes in refractive correction. These results suggest that the association between changes in oblique astigmatism following cataract surgery in older people and increased dizziness,^7^ may be at least partly due to the effects of oblique astigmatic changes on SVV. Dizziness is multifactorial, but impaired vision is a risk factor,^25^ likely through its effect of decreasing postural stability.^26^ In addition, large changes in spectacle correction (and thus magnification) have been shown to change the vestibulo-ocular reflex gain^27^ and could contribute to dizziness. We propose that the effect of oblique astigmatism on verticality perception is also a contributor to the problem. It seems likely that the poor adaptation to spectacles that include cylindrical change, especially in an oblique direction,^1^^,^^2^ in older people is partly because of this effect. Our results provide research evidence to help explain why older adults have difficulty adapting to new spectacles that contain astigmatic changes, whereas younger adults do not. They also help explain why difficulties adapting are greater when the astigmatism is at an oblique axis. They reinforce the importance of eye care clinicians both understanding these effects and taking them into account when prescribing changes in refractive correction.
